# Gene Duplication: The Genomic Trade in Spare Parts

**DOI:** 10.1371/journal.pbio.0020206

**Published:** 2004-07-13

**Authors:** Matthew Hurles

## Abstract

The duplication of genes and their subsequent diversification has had a key role in evolution. A range of fates can befall a duplicated gene

If necessity is the mother of invention, then its father is an inveterate tinkerer, with a large garage full of spare parts. Innovation (like homicide) requires motive and opportunity. Clearly, the predominant ‘motive’ during the evolution of a novel gene function is to gain a selective advantage. To understand why gene duplications represent the major ‘opportunities’ from which new genes evolve, we must first consider what constrains genic evolution.

The vast majority of genes in every genome are selectively constrained, in that most nucleotide changes that alter the fitness of the organism are deleterious. How do we know this? Comparisons between genomes clearly demonstrate that coding sequences diverge at slower rates than non-coding regions, largely due to a deficit of mutations at positions where a base change would cause an amino-acid change. Gene duplication provides opportunities to explore this forbidden evolutionary space more widely by generating duplicates of a gene that can ‘wander’ more freely, on condition that between them they continue to supply the original function.

Susumu Ohno was the first to comprehensively elucidate the potential of gene duplication, in his book *Evolution by Gene Duplication*, published more than 30 years ago (Ohno 1970). The prescience of Ohno's book is highlighted by the fact that his book has almost certainly been cited more times in the past five years than in the first five years after its publication.

## What Is the Evidence for the Importance of Gene Duplication?

The primary evidence that duplication has played a vital role in the evolution of new gene functions is the widespread existence of gene families. Members of a gene family that share a common ancestor as a result of a duplication event are denoted as being paralogous, distinguishing them from orthologous genes in different genomes, which share a common ancestor as a result of a speciation event. Paralogous genes can often be found clustered within a genome, although dispersed paralogues, often with more diverse functions, are also common.

Whole genome sequences of closely related organisms have allowed us to identify changes in the gene complements of species over relatively short evolutionary distances. These comparisons typically reveal dramatic expansions and contractions of gene families that can be related to underlying biological differences. For example, humans and mice differ in their sensory reliance on sight and smell respectively; colour vision in humans has been significantly enhanced by the duplication of an Opsin gene that allows us to distinguish light at three different wavelengths, while mice can distinguish only two. By contrast, a much higher proportion of the large gene family of olfactory receptors have retained their functionality in mice, as compared to humans.

Given the apparent importance of gene duplication for the evolution of new biological functions over all evolutionary timescales, it is of great interest to be able to comprehensively document the duplicative differences that exist between our own species and our closest relatives, the great apes. The study by Fortna et al. (2004) in this issue of *PLoS Biology* identifies over 3% of around 30,000 genes as having undergone lineage-specific copy number changes among five hominoid (humans plus the great apes) species. This is the first time that copy number changes among apes have been assayed for the vast majority of human genes, and we can expect that the biological consequences of the 140 human-specific copy number changes identified in this study will be heavily investigated over the coming years.

## How Do Duplications Arise?

**Figure 1:**
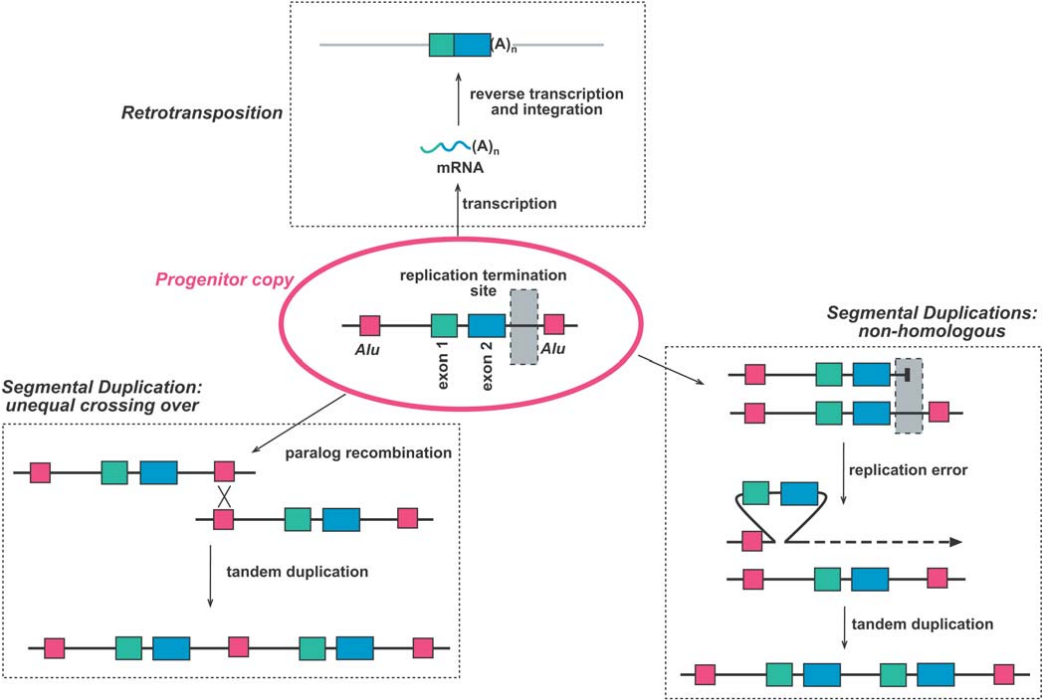
Mechanism of Gene Duplication A two-exon gene is flanked by two *Alu* elements and a neighbouring replication termination site. Recombination between the two *Alu* elements leads to a tandem duplication event, as does a replication error instigated by the replication termination site. Retrotransposition of the mRNA of the gene leads to the random integration of an intron-less paralogue at a distinct genomic location.

The various mechanisms by which genes become duplicated are often classified on the basis of the size of duplication generated, and whether they involve an RNA intermediate (Figure 1).

‘Retrotransposition’ describes the integration of reverse transcribed mature RNAs at random sites in a genome. The resultant duplicated genes (retrogenes) lack introns and have poly-A tails. Separated from their regulatory elements, these integrated sequences rarely give rise to expressed full-length coding sequences, although functional retrogenes have been identified in most genomes.

Tandem duplication of a genomic segment (segmental duplication) is one of the possible outcomes of ‘unequal crossing over’, which results from homologous recombination between paralogous sequences. These recombination events can also give rise to the deletion or inversion of intervening sequences. Recent evidence suggests that the explosion of segmental duplications in recent primate evolution has been caused in part by the rapid proliferation of Alu elements about 40 MYA. Alu elements are derived from the 7SL RNA gene and represent the most frequent dispersed repeat in the human genome, with the approximately 1 million copies of the 300-bp Alu element representing around 10% of the entire genome. The striking enrichment of Alu elements at the junctions between duplicated and single copy sequences implicates unequal crossing over between these repeats in the generation of segmental duplications (Bailey et al. 2003).

The observation of segmental duplication events with no evidence for homology-driven unequal crossing over suggests that segmental duplications can also arise through non-homologous mechanisms. A recent screen for spontaneous duplications in yeast suggests that replication-dependent chromosome breakages also play a significant role in generating tandem duplications, because duplication breakpoints are enriched at replication termination sites (Koszul et al. 2004).

Genome duplication events generate a duplicate for every gene in the genome, representing a huge opportunity for a step-change in organismal complexity. However, genome duplication presents significant problems for the faithful transmission of a genome from one generation to the next, and is consequently a rare event, at least in Metazoa. In principle, genome duplications should be easily identified through the coincident emergence within a phylogeny of many gene families. Unfortunately, this signal is complicated by subsequent piecemeal loss and gain of gene family members. Consequently, there is heated debate over possible ancient genome duplication events in early vertebrate evolution and more recently in teleost fish, both of which must have occurred hundreds of millions of years ago (McLysaght et al. 2002; Van de Peer et al. 2003).

So what are the relative contributions of these different mechanisms? Not all interspersed duplicate genes are generated by retrotransposition. The initially tandem arrangement of segmental duplications can be broken up by subsequent rearrangements. In keeping with this hypothesis, duplicated genes in a tandem arrangement typically represent more recent duplication events (Friedman and Hughes 2003). Recent analyses suggest that 70% of non-functional duplicated genes (pseudogenes) in the human genome result from retrotransposition rather than any DNA-based process (Torrents et al. 2003).

## What Fates Befall a Recently Duplicated Gene?

A duplicated gene newly arisen in a single genome must overcome substantial hurdles before it can be observed in evolutionary comparisons. First, it must become fixed in the population, and second, it must be preserved over time. Population genetics tells us that for new alleles, fixation is a rare event, even for new mutations that confer an immediate selective advantage. Nevertheless, it has been estimated that one in a hundred genes is duplicated and fixed every million years (Lynch and Conery 2000), although it should be clear from the duplication mechanisms described above that it is highly unlikely that duplication rates are constant over time. However, once fixed, three possible fates are typically envisaged for our gene duplication.

Despite the slackened selective constraints, mutations can still destroy the incipient functionality of a duplicated gene: for example, by introducing a premature stop codon or a mutation that destroys the structure of a major protein domain. These degenerative mutations result in the creation of a pseudogene (nonfunctionalization). Over time, the likelihood of such a mutation being introduced increases. Recent studies suggest that there is a relatively narrow time window for evolutionary exploration before degradation becomes the most likely outcome, typically of the order of 4 million years (Lynch and Conery 2000).

During the relatively brief period of relaxed selection following gene duplication, a new, advantageous allele may arise as a result of one of the gene copies gaining a new function (neofunctionalization). This can be revealed by an accelerated rate of amino-acid change after duplication in one of the gene copies. This burst of selection is necessarily episodic—once a new function is attained by one of the duplicates, selective constraints on this gene are reasserted. These patterns of selection can be observed in real data: most recently duplicated gene pairs in the human genome have diverged at different rates from their ancestral amino-acid sequence (Zhang et al. 2003). A convincing instance of neofunctionalization is the evolution of antibacterial activity in the *ECP* gene in Old World Monkeys and hominoids after a burst of amino-acid changes following the tandem duplication of the progenitor gene *EDN* (a ribonuclease) some 30 MYA (Zhang et al. 1998). The divergence of duplicated genes over time can be also monitored in genome-wide functional studies. In both yeast and nematodes, the ability of a gene to buffer the loss of its duplicate declines over time as their functional overlap decreases.

**Figure 2:**
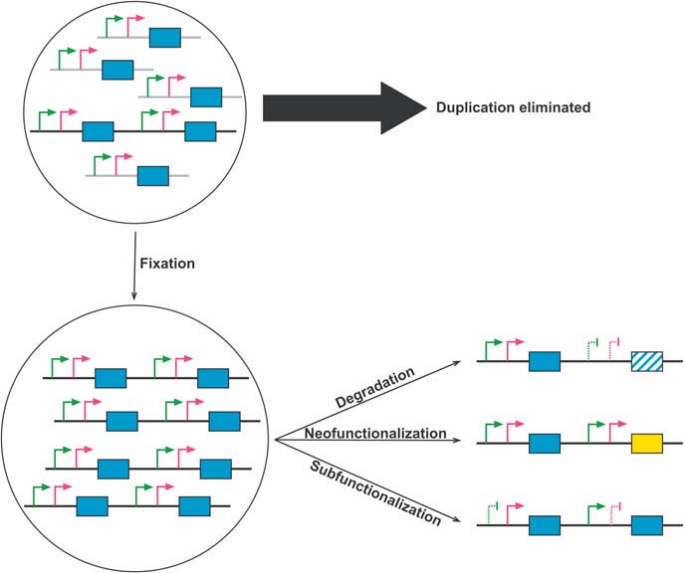
Fates of Duplicate Genes A new duplication in a gene (blue) with two tissue-specific promoters (arrows) arises in a population of single copy genes. Fixation within the population results in a minority of cases. After fixation, one gene is inactivated (degradation) or assumes a new function (neofunctionalization), or the expression pattern of the original gene is partitioned between the two duplicates as one promoter is silenced in each duplicate in a complementary manner (subfunctionalization).

Rather than one gene duplicate retaining the original function, while the other either degrades or evolves a new function, the original functions of the single-copy gene may be partitioned between the duplicates (subfunctionalization). Many genes perform a multiplicity of subtly distinct functions, and selective pressures have resulted in a compromise between optimal sequences for each role. Partitioning these functions between the duplicates may increase the fitness of the organism by removing the conflict between two or more functions. This outcome has become associated with a population genetic model known as the Duplication–Degeneration–Complementation (DDC) model, which focuses attention on the regulatory changes after duplication (Force et al. 1999). In this model, degenerative changes occur in regulatory sequences of both duplicates, such that these changes complement each other, and the union of the expression patterns of the two duplicates reconstitutes the expression pattern of the original (Figure 2).

A recent study by Dorus and colleagues (Dorus et al. 2003) investigated the retrotransposition (since the existence of a human–mouse common ancestor) of one of the two autosomal copies of the *CDYL* gene to Y chromosome (forming *CDY*). In the mouse, both *Cdyl* genes produce two distinct transcripts, one of which is expressed ubiquitously while the other is testis-specific. By contrast, in humans both *CDYL* genes produce a single ubiquitously expressed transcript, and *CDY* exhibits testis-specific expression. As *CDY* is a retrogene (see above) that has not been duplicated together with its ancestral regulatory sequences, it is clear that the DDC model is not the only route by which to achieve spatial partitioning of ancestral expression patterns.

Subfunctionalization can also lead to the partitioning of temporal as well as spatial expression patterns. In humans, the β-globin cluster of duplicated genes contains three genes with coordinated but distinct developmental expression patterns. One gene is expressed in embryos, another in foetuses, and the third from neonates onwards. In addition, coding sequence changes have co-evolved with the regulatory changes so that the O_2_ binding affinity of haemoglobin is optimised for each developmental stage. This coupling between coding and regulatory change is similarly noted at a genomic level when expression differences between many duplicated genes pairs are correlated with their coding sequence divergence (Makova and Li 2003).

## Other Evolutionary Consequences of Gene Duplication

If duplication results in the formation of a novel function as a result of interaction between the two diverged duplicates, which of the above categories of evolutionary outcome does this innovation fall into? Not all new biological functions resulting from gene duplications can be ascribed to individual genes. Protein–protein interactions often occur between diverged gene duplicates. This is especially true for ligand–receptor pairs, which are often supposed to coevolve after a gene duplication event, and thus progress from homophilic to heterophilic interactions. This emergent function of the new gene pair does not fit comfortably into any of the scenarios outlined above: both genes are functional yet neither retains the original function, nor has the original function been partitioned. This mode of ‘duplicate co-evolution’ is likely to be especially prevalent in signalling pathways.

**Figure 3:**
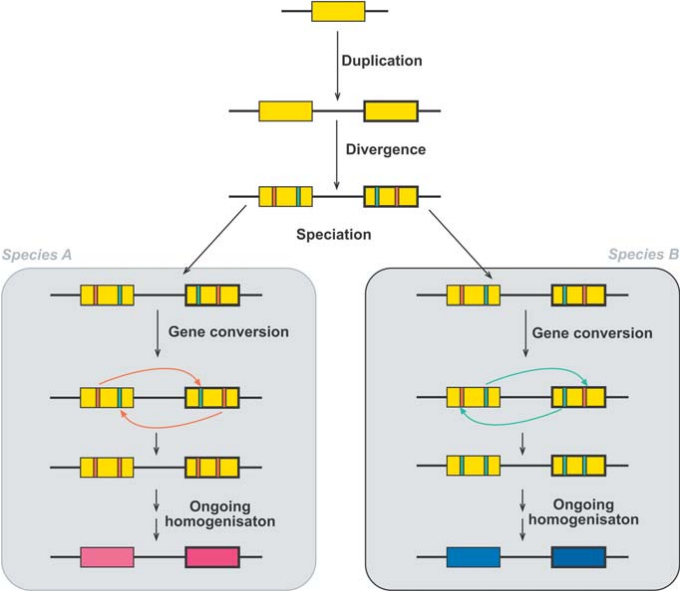
Concerted Evolution Different gene conversion events homogenize minimally diverged duplicate genes in each daughter species (A and B), with the result that while paralogues are highly similar, orthologues diverge over time.

Earlier, we saw that homologous recombination between paralogous sequences can result in rearrangements, including tandem duplications. Such recombination events need not cause rearrangements, but can also result in the nonreciprocal transfer of sequence from one paralogue to the other—a process known as gene conversion. Gene conversion homogenizes paralogous sequences, retarding their divergence, and consequently obscuring their antiquity. This leads to the observation of ‘concerted evolution’ whereby duplicates within a species can be highly similar and yet continue to diverge between species (Figure 3). Once gene duplicates have diverged sufficiently so that they differ in their functionality (or non-functionality), gene conversion events can become deleterious—for example, by introducing disrupting mutations from a pseudogene into its functional duplicate. A substantial proportion of disease alleles in Gaucher disease result from the introduction of mutations into the glucocerebrosidase gene from a tandemly repeated pseudogene (Tayebi et al. 2003). These kinds of recombinatorial interactions only occur between paralogues that are minimally diverged. Thus, while selective interactions and functional overlap between duplicates declines relatively slowly over evolutionary time, the potential for recombinatorial interactions between paralogues is relatively short-lived.

For some genes, duplication confers an immediate selective advantage by facilitating elevated expression, or as Ohno put it, ‘duplication for the sake of producing more of the same’. This has clearly been the case for histones and ribosomal RNA genes. In this scenario, gene conversion is of potential benefit in maintaining homogeneity between copies. Certainly both histone and rDNA genes are commonly found in arrays of duplicates: structures that facilitate array homogenization by both gene conversion and repeated unequal crossing over.

Mechanisms of segmental duplication are oblivious to where genes begin and end, and so are additionally capable of duplicating parts of genes or several contiguous genes. The intragenic duplication of individual exons or enhancer elements also presents new opportunities for the evolution of new functions or greater regulatory complexity.

## Conclusions

The likelihood that newly duplicated genes will both remain functional clearly relates to their inherent potential to undergo subfunctionalization or neofunctionalization. Under the DDC model, greater regulatory complexity bestows greater potential for subfunctionalization (Force et al. 1999), whereas neofunctionalization is more likely to occur in genes that are necessarily rapidly evolving, such as those involved in reproduction, immunity, and host defence (Emes et al. 2003). This is not to say that these biases are deterministic, there are plenty of ‘successful’ gene family clusters that contain associated pseudogenes.

Duplicate gene evolution has most likely played a substantial role in both the rapid changes in organismal complexity apparent in deep evolutionary splits and the diversification of more closely related species. The rapid growth in the number of available genome sequences presents diverse opportunities to address important outstanding questions in duplicate gene evolution. For those interested in patterns of selection following duplication, the transient nature of the evolutionary window of opportunity following duplication will focus attention on recently duplicated genes. In this regard it will be important to document copy number variation not only among species, as Fortna et al. have, but within species as well. In addition, it has been, and will continue to be, a lot easier to identify copy number changes between genomes than it is to identify their biological consequences (if any). Extensive functional studies targeted at duplicated genes are required if we are to more fully understand the range of evolutionary outcomes. Moreover, collaborations between the proteomics and evolutionary genetics communities would facilitate investigation of the potential role of gene duplication during the evolution of the protein–protein and cell–cell interactions that are fundamental to the biology of multicellular organisms.
